# Influential Insider: *Wolbachia*, an Intracellular Symbiont, Manipulates Bacterial Diversity in Its Insect Host

**DOI:** 10.3390/microorganisms9061313

**Published:** 2021-06-16

**Authors:** Morgane Ourry, Agathe Crosland, Valérie Lopez, Stéphane A. P. Derocles, Christophe Mougel, Anne-Marie Cortesero, Denis Poinsot

**Affiliations:** 1 Institut de Génétique, Environnement et Protection des Plantes (IGEPP), INRAE, Agrocampus Ouest, Université de Rennes, F-35650 Le Rheu, France; christophe.mougel@inrae.fr; 2 Institut de Génétique, Environnement et Protection des Plantes (IGEPP), INRAE, Agrocampus Ouest, Université de Rennes, F-35000 Rennes, France; crosland.agathe@gmail.com (A.C.); valerie.dolores.lopez@gmail.com (V.L.); stephane.derocles@univ-rennes1.fr (S.A.P.D.); anne-marie.cortesero@univ-rennes1.fr (A.-M.C.); denis.poinsot@univ-rennes1.fr (D.P.)

**Keywords:** cabbage root fly, *Delia radicum*, *Wolbachia*, endosymbiont, *Erwinia*, bacterial communities, network, interactions, antagonism

## Abstract

Facultative intracellular symbionts like the α-proteobacteria *Wolbachia* influence their insect host phenotype but little is known about how much they affect their host microbiota. Here, we quantified the impact of *Wolbachia* infection on the bacterial community of the cabbage root fly *Delia radicum* by comparing the microbiota of *Wolbachia*-free and infected adult flies of both sexes. We used high-throughput DNA sequencing (Illumina MiSeq, 16S rRNA, V5-V7 region) and performed a community and a network analysis. In both sexes, *Wolbachia* infection significantly decreased the diversity of *D. radicum* bacterial communities and modified their structure and composition by reducing abundance in some taxa but increasing it in others. Infection by *Wolbachia* was negatively correlated to 8 bacteria genera (*Erwinia* was the most impacted), and positively correlated to *Providencia* and *Serratia*. We suggest that *Wolbachia* might antagonize *Erwinia* for being entomopathogenic (and potentially intracellular), but would favor *Providencia* and *Serratia* because they might protect the host against chemical plant defenses. Although they might seem prisoners in a cell, endocellular symbionts can impact the whole microbiota of their host, hence its extended phenotype, which provides them with a way to interact with the outside world.

## 1. Introduction

Most endosymbionts are facultative but can contribute greatly to insect host fitness. Such effects include a higher fecundity, resistance to heat shock [1,2], protection against natural enemies [3], or plant defensive mechanisms [4]. While insects host more or less complex microbial communities (named microbiota, mostly present in the gut), only a few studies have focused on how an endosymbiont could affect the rest of the bacterial communities.

*Wolbachia* (α-Proteobacteria: Anaplasmataceae) is a maternally-transmitted facultative endocellular symbiont (i.e., endocytobiont) infecting arthropods and nematodes. It was initially thought to be only present in gonads [5] where it is vertically transmitted from the females into the egg cytoplasm [6], but it is now clear that *Wolbachia* can be found in many somatic tissues [7,8]. In arthropods, *Wolbachia* is widespread: 50 to 75% of species are probably infected by this symbiont [9,10,11]. In these hosts, *Wolbachia* manipulates the host reproduction in order to ensure a high infection rate of female offspring through male feminization, male killing, parthenogenesis, or cytoplasmic incompatibility [9,12]. Several studies have also revealed positive effects of *Wolbachia* on host fecundity or survival [12]. Additionally, *Wolbachia* can modify host learning and memory as well as its feeding, sleeping, locomotive, and aggressive behaviors [13]. Despite the extensive literature demonstrating *Wolbachia’s* effects on their host phenotypes, little is known about whether and how much *Wolbachia* can affect their host microbiota. However, the impact of the microbiota on many functions of the host is now clear [2,14,15,16]; accordingly, being able to modify this bacterial community would provide *Wolbachia* with a lever to influence the host.

To our knowledge, only seven studies have assessed the effect of *Wolbachia* on its host bacterial communities: in *Drosophila melanogaster* [17,18], in *Aedes aegypti* [19] and *Anopheles stephensi* [20] mosquitoes, in *Armadillidium vulgare* [21], in several tick species [22], and in spider mites [23]. *Wolbachia* decreased the bacterial diversity of *D. melanogaster* and of ticks [18,22] but not of mosquitoes [19,20] nor spider mites [23]. *Wolbachia* modified the relative abundance of bacteria in isopods [21] and *A. aegypti* [19], but also determined the presence or absence of other bacteria [21]. More hosts obviously need to be investigated before we understand how much and by which mechanism *Wolbachia* influences whole bacterial communities.

Because *Wolbachia* influences its host reproduction and gender, whether *Wolbachia* has the same influence on female and male bacterial communities is an important question. Dittmen and Bouchon (2018) investigated this issue but were not able to conclude because host gender was a confounding factor in their study: there was no way to determine whether *Wolbachia*-infected isopods were genetically female or male because of male feminization and rare uninfected females [21]. So far, two cases of insect male feminization were reported in Lepidopterans [24] and Hemipterans [25], but none in Dipterans [26].

The cabbage root fly (*Delia radicum*, Diptera: Anthomyiidae) is a root herbivore of Brassicaceous species such as cabbages and turnips [27]. Its larvae develop by feeding and tunneling inside roots while the adult flies rely on chemical signals emitted by leaves to select the most favorable host plant to lay eggs [28,29]. *Wolbachia* is a facultative endosymbiont of *D. radicum*, where in the field some populations are infected and some populations are *Wolbachia*-free [30]. A laboratory study showed that *Wolbachia* only moderately influences host life history traits in *D. radicum* with no obvious positive or negative outcome as a result [31]. The bacterial communities of *D. radicum* have been described both in the presence [32] and in the absence of *Wolbachia* [33]. A comparison of these studies seems to show a difference in bacterial diversity and a twofold decrease of the Shannon index in presence of *Wolbachia*. However, these separate works used different sequencing techniques and did not allow direct measurement of if and how much *Wolbachia* shaped the bacterial communities of *D. radicum*. The present study aims to fill this gap.

Here, we measured the impact of *Wolbachia* on the bacterial communities of *D. radicum* adult flies. We created two comparable *Wolbachia*-free (“W−”) and infected (“W+”) lines. We then sequenced the bacterial communities of individuals of both sexes using high-throughput DNA sequencing (Illumina MiSeq) of a 424 bp fragment of the 16S rRNA gene, frequently used in sequencing studies that also identifies taxa to the genus level. Following previous studies [17,18,21,22], we hypothesized that *Wolbachia* decrease bacterial community diversity, modify its structure, and upset bacterial dominances by altering both bacterial presence and abundances. Our results show that *Wolbachia* infection decreased the diversity and shifted the structure and composition of adult *D. radicum* bacterial communities, revealing negative and positive interactions between *Wolbachia* and the resident bacteria.

## 2. Materials and Methods

### 2.1. Fly Population and Line Creation

The *D. radicum* strain used in the experiment came from pupae collected in 2014 in a broccoli (*Brassica oleracea*) field at Le Rheu (Brittany, France, 48°07′16″ N, 1°47′41″ O). After emergence, these flies were reared in a climatic chamber (21 ± 1 °C, 60 ± 10% RH, L16:D8) on rutabaga roots (*Brassica napus* subsp. *rapifera*) as previously described [34]. For preliminary tests, flies from this rearing population were repeatedly sampled over three years (i.e., approximately 30 generations) to assess their infection status by PCR as described below, which revealed that the population was polymorphic for *Wolbachia* infection. To establish separate lines according to infection status, 68 inseminated females (8 to 15 days old) were separated to lay eggs individually, after which their *Wolbachia* infection status was tested by PCR. This revealed 39 *Wolbachia-*infected and 29 *Wolbachia*-free females. Their offspring were then pooled according to their mother’s status, establishing the first generation of the W+ and W− lines respectively [31]. After oviposition of each generation, females were collected to assess the presence of *Wolbachia* and confirm its transmission. These steps thus allowed the creation of a *Wolbachia*-free (“W−”) and a *Wolbachia*-infected (“W+”) population sharing the same genetic background, which was also used and described in our previous work [31].

The 47 flies used in our experiment came from the sixth generation (i.e., since the creation of both lines) and were collected alive and fed between 7 and 10 days after emergence: 11 W− females, 12 W− males, 12 W+ females, 12 W+ males. They were stored in 96% ethanol at –20 °C until further analysis.

### 2.2. Molecular Analyses of Fly Bacterial Communities

DNA was extracted from the females used to create the lines and from the W+ and W− 6th generation flies used in our experiment by “salting-out” as previously described [33].

To confirm the presence of *Wolbachia* in females creating the lines and in our 6th generation flies, we performed PCR using primers FbpA_F1 (5′-GCTGCTCCRCTTGGYWTGAT-3′) and FbpA_R1 (5′-CCRCCAGARAAAAYYACTATTC-3′) [10] in conditions previously described [31].

To analyze *D. radicum* bacterial communities, a 424 bp fragment of the V5–V7 region of the bacterial 16S rRNA gene was amplified using the primers 799F (5′-AACMGGATTAGATACCCKG-3′) and 1223R (5′-CCATTGTAGTACGTGTGTA-3′). PCR amplification, library preparation, Illumina MiSeq sequencing (i.e., 2 × 300 bases paired-end version), demultiplexing, and barcode suppression in our 6th generation flies were performed by GenoScreen (Lille, France) as previously described [33].

### 2.3. Bioinformatical and Statistical Analyses

Analyses were performed using the R software [35]. The dada2 workflow (v. 1.8, “dada2” R package; [36]), based on Divisive Amplicon Denoising Algorithm (“DADA”) was adapted (trimming, error rate learning, and sequence length inspection) as previously described [33] and used on the 47 samples to obtain an amplicon sequence variant (ASV) table. ASV identified fine-scale variations down to the genus level compared to operational taxonomic units.

#### 2.3.1. Data Cleaning

Raw data were handled as previously described [33] using the R software [35], with the “phyloseq” and “microbiome” packages [37,38] and the “ggplot2” package for the construction of the plots [39]. Data were rarefied at a sample size of 3500, then expressed in per mille proportions and filtered from the proportions lower than 1/1000.

#### 2.3.2. Bacterial Diversity and Structure

The alpha diversity of the bacterial communities was analyzed using rarefied and proportion-expressed data by calculating the number of observed ASVs, the Shannon index, and the evenness (i.e., Pielou index). The effect of *Wolbachia*, the gender of individuals, and the interaction between both factors on the alpha diversity was tested using linear models (type II ANOVA, F-test).

The beta diversity of the bacterial communities was assessed using rarefied, proportion-expressed and filtered data. Beta diversity analyses were performed on both presence/absence data and abundance data. The community structure was analyzed: using (i) the Hellinger distance and a transformation-based redundancy analysis (tb-RDA) on the presence/absence data and (ii) the Bray–Curtis dissimilarity matrix and a distance-based redundancy analysis (db-RDA) on the abundance data. The effects of *Wolbachia*, the gender, and their interaction were tested on each type of community structure with a type II permutation F-test for constrained multivariate analyses. Data were plotted using the “MVA.plot” function from the “RVaideMemoire” package [40].

#### 2.3.3. Bacterial Taxonomy

Differences in the presence (i.e., frequency) and in the abundance of bacterial genera between the *Wolbachia* treatment, the gender of individuals, and the interaction of both factors were tested. For the presence/absence data, the frequency of each genus was tested with a generalized linear model (GLM, binomial error, logit link function) and a likelihood-ratio test. For abundance data, we built differential heat trees with the “metacoder” package [41] to visualize community data at every taxonomical scale (i.e., from phylum to genus). On the heat trees, the default Wilcoxon rank-sum test and *p* values corrected with the “False Discovery Rate” (FDR) were used to assess the effects of *Wolbachia*, including and excluding the effect of the gender of the individuals.

The presence of specific or shared taxa between W− and W+ treatments, also including and excluding the effect of the gender of the individuals, was assessed using another type of heat trees on the presence/absence table as previously described [33]. Thus, only taxa present in all flies of one or two compared treatments were colored on the differential heat trees.

#### 2.3.4. Network Analysis

A network analysis was conducted with the SparCC method (Sparse Correlations for Compositional data) and the thresholds of –0.5 and 0.5 for the Pearson correlation coefficient. To determine how *Wolbachia* is correlated to other bacterial genera, we used the “SpiecEasi” [42], “igraph” [43], and “qgraph” [44] packages, as previously described [45]. This analysis was performed on the whole rarefied dataset (W− and W+ individuals together), as well as on W− and W+ samples separately. Several network features were calculated:

- the small-worldness score (i.e., if score is higher than 1, the network is considered to be a small-world type which “most nodes are accessible to every other node through a relatively short path” [45]);
- the modularity (i.e., the density of the network connections within certain groups of nodes and sparse connections);
- the number of clusters (using the walktrap algorithm);
- the link density (i.e., the proportion of possible links between taxa) obtained using the “normalized = TRUE” argument of the “degree” function (“igraph” package);
- the hubbiness score (i.e., the probability for a taxa to be a hub genus);
- the number of articulation points (i.e., nodes, also called vertices, which removals increased the number of connected components).

## 3. Results

After sequencing, the mean total read depth was 17,961 ± 446 for *Wolbachia*-free (“W−”) females; 18,584 ± 1505 for W− males; 18,640 ± 752 for *Wolbachia*-infected (“W+”) females, and 18,519 ± 712 for W+ males. Overall, 3206 ASVs were detected. After the rarefaction step, 2081 taxa and 1 sample (W− male) were eliminated; 702 ASVs remained after the proportion-expressed and filtration steps.

### 3.1. Assessment of Wolbachia

The *Wolbachia* genus was confirmed to be absent from all W− female and male samples while it was detected in 100% of the W+ female and 83% of the W+ male samples (Figure S1). However, one female and two male W+ samples had a very low abundance of *Wolbachia* (<3‰) and were thus removed from the dataset before further analysis, along with ASVs only detected in these three samples.

### 3.2. Diversity and Structure of the Fly Bacterial Communities

**Figure 1:**
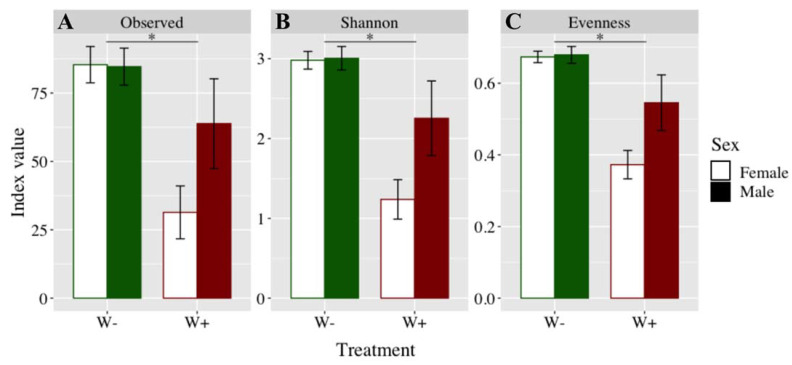
Bacterial alpha diversity of the *Wolbachia*-free (“W−” in green) and infected (“W+” in red) lines. Mean values, standard errors, *: *p* < 0.001.

Alpha diversity, represented by the number of observed ASVs (F_1.39_ = 13.49; *p* = 0.001), the Shannon index (F_1.39_ = 22.05; *p* < 0.001), and the evenness (F_1.39_ = 25.00; *p* < 0.001), was significantly reduced by *Wolbachia* (Figure 1). The number of observed ASVs (F_1.39_ = 2.25; *p* = 0.141), the Shannon index (F_1.39_ = 3.59; *p* = 0.098), and the evenness (F_1.39_ = 3.95; *p* = 0.098) were not influenced by gender. The *Wolbachia* × gender interaction did not affect the number of observed ASVs (F_1.39_ = 2.59; *p* = 0.115), the Shannon index (F_1.39_ = 3.41; *p* = 0.108), and the evenness (F_1.39_ = 3.62; *p* = 0.108).

**Figure 2:**
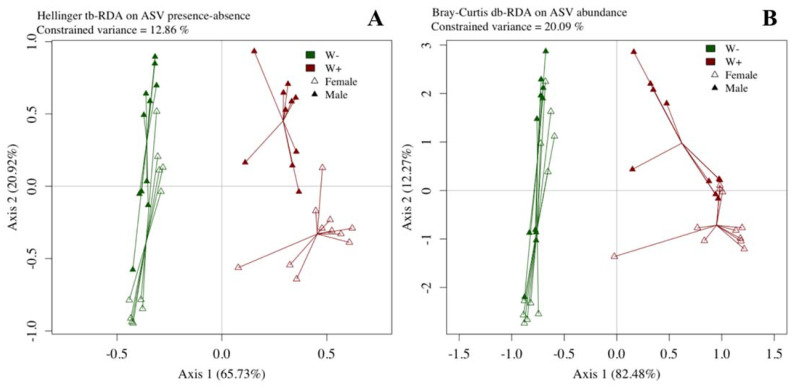
Bacterial community structure of the *Wolbachia*-free (“W−” in green) and infected (“W+” in red) lines.

Beta diversity was affected by *Wolbachia* for both analyses (tb-RDA: F = 3.70; *p* = 0.001, db-RDA: F = 7.90; *p* = 0.001, Figure 2) but not by gender (tb-RDA: F = 1.23; *p* = 0.163, db-RDA: F = 1.26; *p* = 0.246) nor by the *Wolbachia* × gender interaction (tb-RDA: F = 0.80; *p* = 0.708, db-RDA: F = 0.60; *p* = 0.789). Our tb-RDA and db-RDA models explained 12.86% and 20.09% of the constrained variance respectively, with *Wolbachia* explaining 7.02% and 14.23%, gender 2.33% and 0.30%, and their interaction 1.52% and 14.81% of the variance.

### 3.3. Dominant Bacterial Taxa

**Figure 3:**
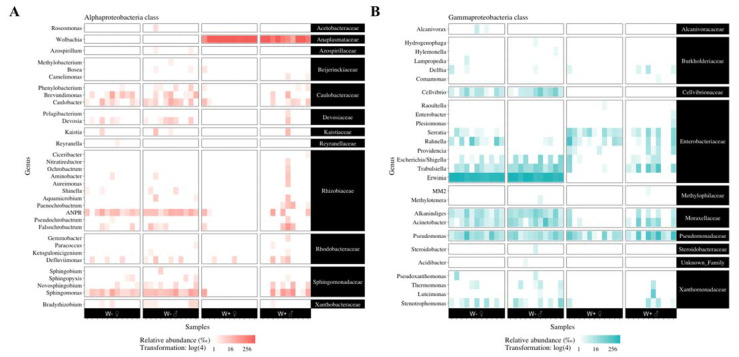
Bacterial genera from the α- (**A**) and γ-Proteobacteria (**B**) present in *Wolbachia*-free (“W−”) and infected (“W+”) lines. The relative abundance is expressed per mille and was log-transformed. ANPR = *Allorhizobium*–*Neorhizobium*–*Pararhizobium*–*Rhizobium*.

The bacterial communities of *D. radicum* were mainly composed of Proteobacteria, followed by the Bacteroidetes, Firmicutes, Gemmatimonadetes, and Verrucomicrobia phyla (Figure S2). In the absence of *Wolbachia*, *Erwinia* largely dominated the communities (Table S1), with a few γ-proteobacterial Enterobacteriaceae (*Trabulsiella*), Moraxellaceae (*Alkanindiges* and *Acinetobacter*), and Cellvibrionaceae (*Cellvibrio*) (Figure 3).

A total of 660 ASVs were detected (Table S2), corresponding to 89 different genera (Table S1). Six genera dominated relative abundances: *Wolbachia* (8 ASVs), *Erwinia* (12 ASVs), *Pseudomonas* (74 ASVs, γ-Proteobacteria class), *Staphylococcus* (20 ASVs, Bacilli class), *Sphingobacterium* (84 ASVs, Bacteroidia class), and *Trabulsiella* (3 ASVs, γ-Proteobacteria class) in a decreasing order. There were 72 and 63 genera present in W− and W+ samples respectively.

### 3.4. Influence of Wolbachia on Other Bacteria

**Figure 4:**
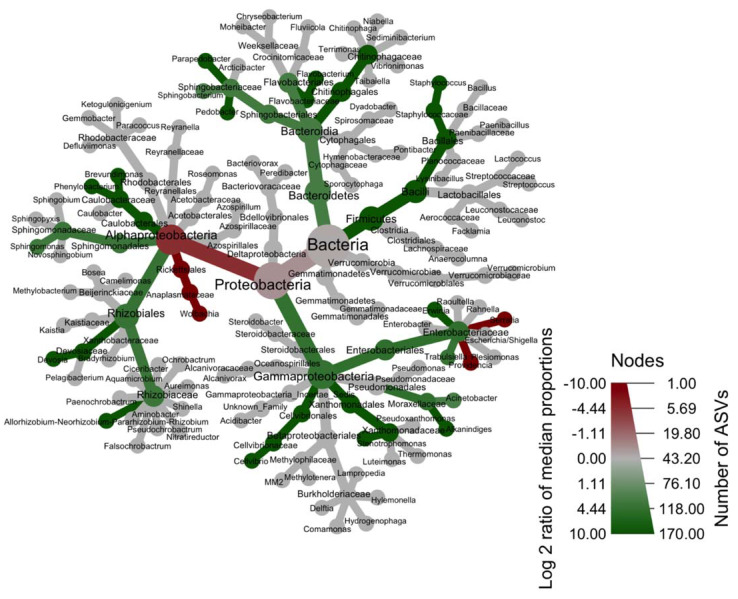
Heat trees comparing taxa relative abundances between *Wolbachia*-free and infected lines. The color of each taxon represents the log-2 ratio of median relative abundances observed for each treatment. Only significant differences are colored. Taxa colored in green are enriched in *Wolbachia*-free flies while taxa colored in red are enriched when *Wolbachia* are present.

The presence of *Wolbachia* significantly increased the frequency and the relative abundance of *Providencia* and *Serratia* but decreased the frequency of 26 genera and the abundance of 16 genera (Figure 4, Table S1). Two genera were also significantly influenced by gender: *Staphylococcus* was more frequent in females and *Novosphingobium* in males. When comparing bacterial abundances between gender within the same treatment, we found no difference for W+ and only few differences for W− flies: only *Staphylococcus* and *Rahnella* were more abundant in W− females (Figure S3).

Moreover, the Enterobacteriaceae family was the only taxon shared by both sexes of both W− and W+ treatments (Figure S4A). Seven bacterial genera (i.e., the γ-proteobacterial *Erwinia*, *Trabulsiella*, *Pseudomonas*, *Acinetobacter*, *Alkanindiges*, *Cellvibrio,* and the α-proteobacterial *Sphingomonas*) were shared between all W− female and male flies, while *Wolbachia* itself was the only genus shared by all W+ flies of both sexes (Figure S4B). In addition to *Wolbachia*, *Serratia* was always present, but only in W+ females.

**Figure 5:**
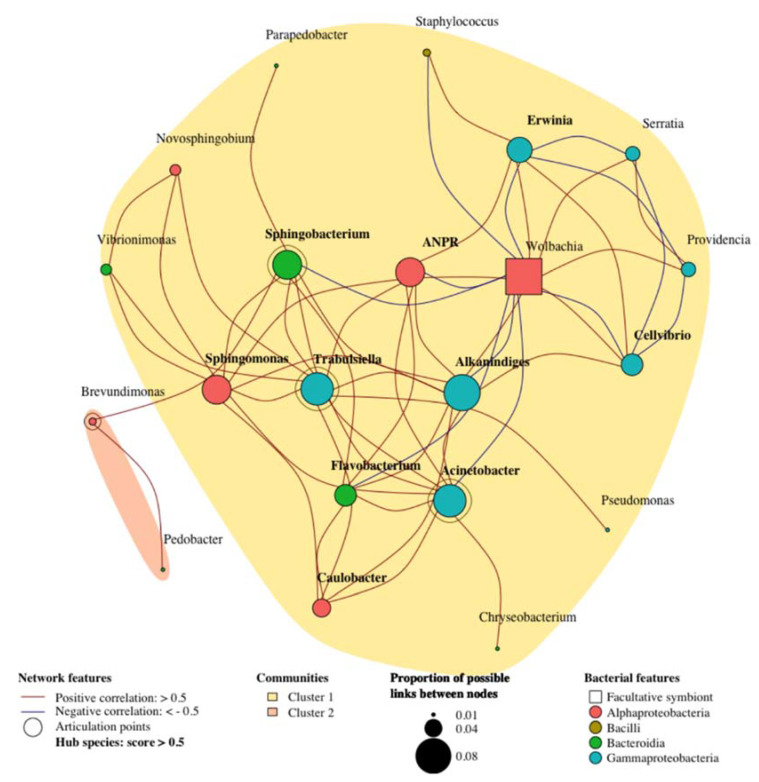
Largest subnetwork of bacterial communities (genus level) associated to *Wolbachia*-free and infected lines altogether. ANPR = *Allorhizobium–Neorhizobium–Pararhizobium–Rhizobium*.

A network analysis of the whole dataset showed that the bacterial communities formed a small-world network, with a small-worldness score of 1.32 and that only a subset of bacteria were connected to each other as the network modularity value was of 0.035, thus rather low (Figure S5). The largest subnetwork (Figure 5) contained two clusters of 19 and 2 bacterial genera. The subnetwork was characterized by the presence of 10 hub bacteria (ANPR, *Caulobacter*, *Sphingomonas*, *Flavobacterium*, *Sphingobacterium*, *Acinetobacter*, *Alkanindiges*, *Cellvibrio*, *Erwinia*, *Trabulsiella*) and 4 articulation points (*Brevundimonas*, *Sphingobacterium*, *Acinetobacter,* and *Trabulsiella*). In the network, the most interconnected bacteria was *Wolbachia*, which was significantly and positively correlated to 2 bacteria (*Providencia* and *Serratia*) and negatively correlated to 8 bacteria (*Staphylococcus*, *Flavobacterium*, *Sphingobacterium*, ANPR, *Acinetobacter*, *Alkanindiges*, *Cellvibrio,* and *Erwinia* in bacterial phyla order).

When comparing the W− and W+ datasets separately, both also had bacterial communities forming small-world networks respectively with a score of 3.82 and of 1.32. However, the W+ network had sparser connections than the W− network (modularity: 0.066 and 0.406 respectively). The W− network contained 3 clusters of 13, 4, and 3 bacteria, and was characterized by 7 hub bacteria and 6 articulation points while the W+ network had 2 clusters of 20 and 2 bacteria, with *Wolbachia* being the only hub bacteria and articulation point (Figure S6).

## 4. Discussion

We found that *Wolbachia* infection significantly decreased the diversity of *D. radicum* bacterial communities and modified their structure and composition, these effects being similar in both sexes. Additionally, 8 bacteria were negatively correlated to *Wolbachia* infection (most markedly *Erwinia* but also *Staphylococcus*, *Flavobacterium*, *Sphingobacterium*, ANPR, *Acinetobacter*, *Alkanindiges,* and *Cellvibrio*) while the correlation was positive for *Providencia* and *Serratia* in the bacterial network.

### 4.1. Wolbachia Reduces the Diversity and Modifies the Structure of D. radicum Bacterial Communities

*Wolbachia* infection significantly reduced bacterial diversity in its host by 43% (Shannon index 1.72 vs. 2.99 in *Wolbachia*-free (“W−”) flies) and decreased the modularity of the microbial network. Such a decrease supports our prediction and the ones made in our previous work [33]. Similar diversity values were found previously in W− flies, sequenced in the same batch as our samples [33]. The diversity we found here in our *Wolbachia*-infected (“W+”) flies seems higher than previously described where OTUs was used [32], possibly due to the difference in taxonomy resolution between ASVs and OTUs. These authors also sampled directly in the field [32] while our stock was reared for several years in the laboratory—the difference of bacterial diversity between field and laboratory insect samples is already known [46,47,48]. A lower diversity in W+ hosts was also found in *D. melanogaster* using the Simpson diversity index compared to W− individuals [18].

In our study, *Wolbachia* also changed the community structure of its host microbiota as well as the bacterial network. Among the seven previous similar studies [17,18,19,20,21,22,23], only three analyzed bacterial community structure. Our result is consistent with that found in isopods [21] and spider mites [23] but not with the mosquito study [20], which found that community structure was not affected by *Wolbachia* infection. Apart from host effects, such differences could be due to the method used to obtain W− and W+ individuals; like Dittmer and Bouchon (2018), we used a naturally polymorphic stock to obtain W− and W+ lines [21] while Chen et al. (2016) used a tetracycline treatment [20] which is bound to impact bacterial communities [33].

### 4.2. Wolbachia Changes the Composition of the Bacterial Communities

We found that in W− individuals, γ-Proteobacteria dominate the *D. radicum* microbial community (mostly through *Erwinia)*. Similarly, *Erwinia* was previously detected, with other Enterobacteriaceae, at the larval stage of W− *D. radicum* but it was barely present in our previous study on W− adult flies [33]. Such difference with the present study could be due to the origin of the fly population (collected in 2014 here vs 2015 [33]) and due to *Erwinia* abundance varying in the field because of climatic conditions and differently acquired through feeding [49].

Interestingly, the prevalence of *Wolbachia* has been recently assessed in *D. radicum* populations from different fields in Brittany (France) and the level of natural infection was rather low, ranging from 0 to 10% [30]. Based on this observation, it could be considered that the W− microbiota may be the “normal” or native microbiota of this host.

In the presence of *Wolbachia*, the number of bacterial genera decreased similarly to results from previous studies [32,50]. However, despite *Wolbachia* presence, a couple of bacteria were also very abundant in those previous studies: the α-proteobacterial *Gluconacetobacter* accounted for a fifth of the total number of reads [32] while several γ-Proteobacteria (*Buttiauxella*, *Morganella*, *Providencia*, and *Rahnella*) were abundant in *D. radicum* adult flies [50]. Here, *Wolbachia* was the only abundant bacteria in W+ flies and the difference with the two previous studies may come from the fly population origin or from molecular protocols (PCR and/or sequencing).

### 4.3. Wolbachia Influence Male and Female Bacterial Communities Equally

We found that the insect gender did not strongly shape *D. radicum* bacterial communities and that the presence of *Wolbachia* did not induce major changes between female and male microbiota. Recent studies on the cabbage root fly found similar results, with no gender effect at all in W+ hosts [50] and a small gender effect only in W− ones [33]. The weak gender effect we found is consistent with *Wolbachia* being similarly abundant in females and males (Figure S1).

### 4.4. Negative Interactions with Wolbachia

We showed that *Wolbachia* was negatively correlated to eight bacteria, which were differently impacted. Here, *Erwinia* was the most impacted as it dominated the community in the absence of *Wolbachia* but could not be detected in its presence. *Erwinia* are multi-faceted bacteria which can be either beneficial or harmful to their insect hosts. For instance, *Erwinia* can improve host nutrition in the olive fly *Bactrocera oleae* by providing it with nitrogen through nitrogen fixation and recycling of non-essential amino acids and urea [51] or by producing cellulase, an enzyme that degrades the cellulose of plant cell walls [52]. An *Erwinia* species from *D. radicum* larval gut is highly resistant to isothiocyanate compounds [53], which are toxic to herbivorous insects [54] and are emitted by plants as a defense [55]. While *Erwinia* protect *Lutzomyia longipalpis* sandflies against parasitism by *Leishmania* [56], they are on the contrary pathogenic for their pea aphids hosts *Acyrthosiphon pisum* [57,58]. Similarly to our study, *Wolbachia* and *Erwinia* seem strikingly unable to cohabit in pea aphids [59] or in *D. radicum* adult flies [32,50]. The apparent antagonism we found between *Wolbachia* and *Erwinia* could mean that *Erwinia* is pathogenic to *D. radicum* and that *Wolbachia* is selected to protect its host—like it does in mosquitoes (*Anopheles* sp.) against *Plasmodium* sp., the agent of the malaria disease [60] or in some *Drosophila* species where it protects against viruses [3]. In support of this hypothesis, a case of *Wolbachia* protection against the pathogenic *Erwinia carotorovra* was demonstrated in mosquitoes (*Aedes aegypti*)—though not in drosophila [61].

Interestingly, an *Erwinia*-like symbiont decreases *Wolbachia* load in the giant scale *Coelostomidia wairoensis* and the authors hypothesize a competition for resources between the two bacteria [62]. Because *Wolbachia* is intracellular, such a competition would seem to require that the *Erwinia*-like symbiont also lives inside its host cells. This might be the case since a phylogenetic study demonstrated that *Erwinia* is closely related to *Buchnera*, the aphid obligate intracellular symbiont [63]. In the olive fly, *Erwinia* is indeed intracellular at the larval stage (in the mycetoma, i.e., midgut gastric ceca) and extracellular only at the adult stage (in the esophageal bulb) [64]. A similar competition hypothesis was proposed in mosquitoes where *Wolbachia* and *Asaia* exclude one another in the reproductive organs where they may compete for the same anatomical niche and infection route toward the next generation [65]. Our observations would therefore warrant further examination of the precise cellular localization of *Erwinia* in larval and adult *D. radicum*.

The abundance of the seven other bacteria negatively correlated to *Wolbachia* infection was far less drastically impacted than that of *Erwinia*, and these bacteria often play positive roles in their insect hosts. The Bacilli *Staphylococcus* reduces parasitism by *Leishmania* in sandflies [56] and also frequently occurs in the order of Lepidoptera, where it may overcome plant protease inhibitor defenses [14]. The Bacteroidia *Flavobacterium* helps digesting plant material by producing cellulase in three phytophagous insect species [52] while *Sphingobacterium* induces resistance against fungal infection in *Delia antiqua* larvae [66]. Although the α-proteobacterial ANPR or *Rhizobium* is usually known as a nitrogen-fixing bacterium living in symbiosis with legume roots [67], it has also been detected in insects [68], where a gene associated to nitrogen fixation was revealed in ants that host Rhizobiales [69]. The γ-proteobacterial *Acinetobacter* improves both its host nutrition and defense by scavenging nitrogen [70], producing cellulase [52], and resisting and degrading plant defenses, respectively in *D. radicum* [53] and in the gypsy moth [71]. *Alkanindiges* is not commonly found in insects but was detected in ants [72] while *Cellvibrio* was first obtained from field soil in Japan but was also detected in a beetle species [73]; the latter bacterial genus is known to degrade polysaccharides such as cellulose [74] which would help the host digesting plant material.

Our results agree with previous studies where *Wolbachia* was found to compete with insect microbiota [75] and also to be a highly interconnected taxon in bacterial networks, where it seems to exclude most bacteria such as *Acinetobacter* in mosquitoes [46] and to decrease the number of hub species and articulation points here. Moreover, all the negatively correlated bacteria we found were absent from *Wolbachia*-infected *D. radicum* flies in the previous studies using that host [32,50]. However, it remains to be demonstrated if (and how) *Wolbachia* truly interacts with these bacteria; however convergent, the observations and correlations obtained here or from the literature are suggestive but still insufficient to establish a causal link.

### 4.5. Positive Interactions with Wolbachia

We demonstrated that *Wolbachia* was positively correlated to *Providencia* and *Serratia*. The γ-proteobacterial *Providencia* is resistant to plant defense isothiocyanates [53] and might provide protection to the host gut, where it might also reduce parasite abundance [56]. *Serratia* has various functions that could clearly benefit the host—such as producing cellulase [52], resisting plant defenses [53], promoting the host resistance against parasites [56] and fungi [66]—but it can also be entomopathogenic [76].

So far, interactions involving *Wolbachia* have mainly been studied to assess how *Wolbachia* itself influences the host phenotype or protects the host against pathogens. Few studies have attempted to determine how *Wolbachia* combined with other bacteria might benefit their common host. Similarly to our study, *Providencia* and *Serratia* have been previously found to be simultaneously present in *D. radicum* bacterial communities [33,53] even when adult flies were infected by *Wolbachia* [50]. While an exclusion of *Serratia* by *Wolbachia* was observed in mosquitoes [46], a co-occurrence was found on the contrary in six *Drosophila* species [77] and in Hormaphidinae and pea aphids [59,78]. The positive correlation we found suggests that *Wolbachia* may benefit from the persistence of *Providencia* and *Serratia,* which indicates that the effect of the latter two genera is probably beneficial (and at least not pathogenic) for the *D. radicum* host. Such correlations call for further studies demonstrating actual beneficial interactions.

### 4.6. Bacterial Switch, Yet the Same Host Performance

Although *Wolbachia* infection strongly modifies the *D. radicum* microbiota, the flies seem to perform well nevertheless. In a study specifically assessing the impact of *Wolbachia* infection on *D. radicum* fitness, opposite but minor effects compensated one another and the fitness of W− and W+ individuals did not differ significantly as a result [31]. An unaffected performance is expected if different bacteria play the same roles in both W− and W+ flies (functional redundancy), which would ensure the proper development of the insect host through maintained nutrition or defense. While the ability of *Wolbachia* to protect some hosts against pathogens has been recognized, its positive impact on host nutrition is less known [79], although it has been shown to synthesize B vitamins, improving host reproduction in the bedbug *Cimex lectularius* [80]. Here, an hypothesis would be that *Erwinia* and the other bacteria negatively correlated to *Wolbachia* infection contribute to *D. radicum* nutrition and resistance against plant defenses and pathogens in W− flies, while *Wolbachia* and the bacteria positively correlated to its abundance fulfill these roles as efficiently in W+ flies, resulting in similar performances in both treatment (see Table 1 for supporting information). However, we did not study bacterial functions but taxonomy, down to the genus level, and different species from the same genus may hold different functions. Whether any of these bacteria are indeed involved in *D. radicum* metabolism or protection against plant defenses remains to be demonstrated through the assessment of bacterial species and gene expression (i.e., functions).

## 5. Conclusions

Our study demonstrates and quantifies a strong *Wolbachia* impact on its *D. radicum* host microbiota. It highlights the potential importance for the host of microbial interactions taking place within its microbiota. However, our approach was correlative; more direct evidence will be required to identify the molecular mechanisms (e.g., gene expression) underlying such host–symbiont and microbial interactions at the species level. Considering that the effects of microbiota on host extended phenotypes have been largely recognized in many taxa, *Wolbachia* most probably indirectly modifies its host extended phenotype whenever it is present (independently from well-known reproductive effects such as cytoplasmic incompatibility or feminization). Furthermore, since most of the microbiota is found in the gut, which is connected to the environment at both ends through oral secretions and feces, infection by *Wolbachia* can also modify signals unwittingly sent by the host in its environment [30]. Our results indicate that the microbiota could be a lever on which intracellular symbionts can pull to influence the outside world.

## Figures and Tables

**Table 1:** Potential functional redundancy between bacteria of *Wolbachia*-free (“W−”) and infected (“W+”) flies.

	Genus	Nutrition	Plant Defense Resistance or Degradation	Pathogen Resistance
W− flies	*Erwinia* ^γ^	Cellulase synthesis [52] Nitrogen fixing and recycling [51]	Isothiocyanates [53]	Yes [56]
	Staphylococcus ^†^	-	Protease inhibitors [14]	Yes [56]
	*Flavobacterium* *	Cellulase synthesis [52]	-	-
	*Sphingobacterium* *	-	-	Yes [66]
	ANPR ^α^	Nitrogen fixation [69]	-	-
	*Acinetobacter* ^γ^	Cellulase synthesis [52] Nitrogen scavenger [70]	Isothiocyanates [53] Phenolic glycosides [71]	-
	*Alkanindiges* ^γ^	-	-	-
	*Cellvibrio* ^γ^	Cellulose degradation [74]	-	-
W+ flies	*Wolbachia* ^α^	B vitamins synthesis [80]	-	Yes [3,60]
	*Providencia* ^γ^	-	Isothiocyanates [53]	Yes [56]
	*Serratia* ^γ^	Cellulase synthesis [52]	Isothiocyanates [53]	Yes [56,66]

^†^: Bacilli; *: Bacteroidia; ^α^: α-Proteobacteria; ^γ^: γ-Proteobacteria. ANPR = *Allorhizobium-Neorhizobium-Pararhizobium-Rhizobium.*

## Data Availability

Raw data sets were deposited in the European Nucleotide Archive database system under the project number PRJEB45534. Accession numbers of fly samples range from ERS6588791 to ERS6588837.

## Supplementary Materials

The following are available online at https://www.mdpi.com/article/10.3390/microorganisms9061313/s1, Figure S1: *Wolbachia* distribution among infected female (♀) and male (♂) flies, Figure S2: Bacterial phyla and classes present in *Wolbachia*-free (“W−”) and infected (“W+”) lines, Figure S3: Heat trees comparing taxa relative abundances between female and male *Wolbachia*-free (“W−”) and infected (“W+”) lines, Figure S4: Heat tree comparing taxa presence (A) between *Wolbachia*-free (“W−”) and infected (“W+”) lines and (B) between female and male W− and W+ lines, Figure S5: Network of bacterial communities associated to *Wolbachia*-free and infected lines altogether, Figure S6: Network and subnetwork of bacterial communities associated to *Wolbachia*-free (“W−”) and infected (“W+”) lines separately, Table S1: Statistical outputs of all genera present in *Wolbachia*-free (“W−”) and infected (“W+”) lines, Table S2: ASV information for *Wolbachia*-free (“W−”) and infected (“W+”) lines.
