# Supplementary material for: Influential Insider: Wolbachia, an Intracellular Symbiont, Manipulates Bacterial Diversity in Its Insect Host
Source: Microorganisms. 2021 Jun 16;9(6):1313. doi: 10.3390/microorganisms9061313 (PMC8234596; doi:10.3390/microorganisms9061313)
Supplement: Supplementary file 1 [file microorganisms-09-01313-s001.zip › Wolbachia_Figure_S1.pdf]

Supporting Information

INFLUENTIAL INSIDER: *WOLBACHIA*, AN INTRACELLULAR SYMBIONT, MANIPULATES BACTERIAL DIVERSITY IN ITS INSECT HOST

Morgane Ourry[1,*], Agathe Crosland[2], Valérie Lopez[2], Stéphane A.P. Derocles[2], Christophe Mougel[1], Anne Marie Cortesero[2] & Denis Poinsot[2]

*** Correspondence:**

Morgane Ourry

morgane.ourry@hotmail.fr

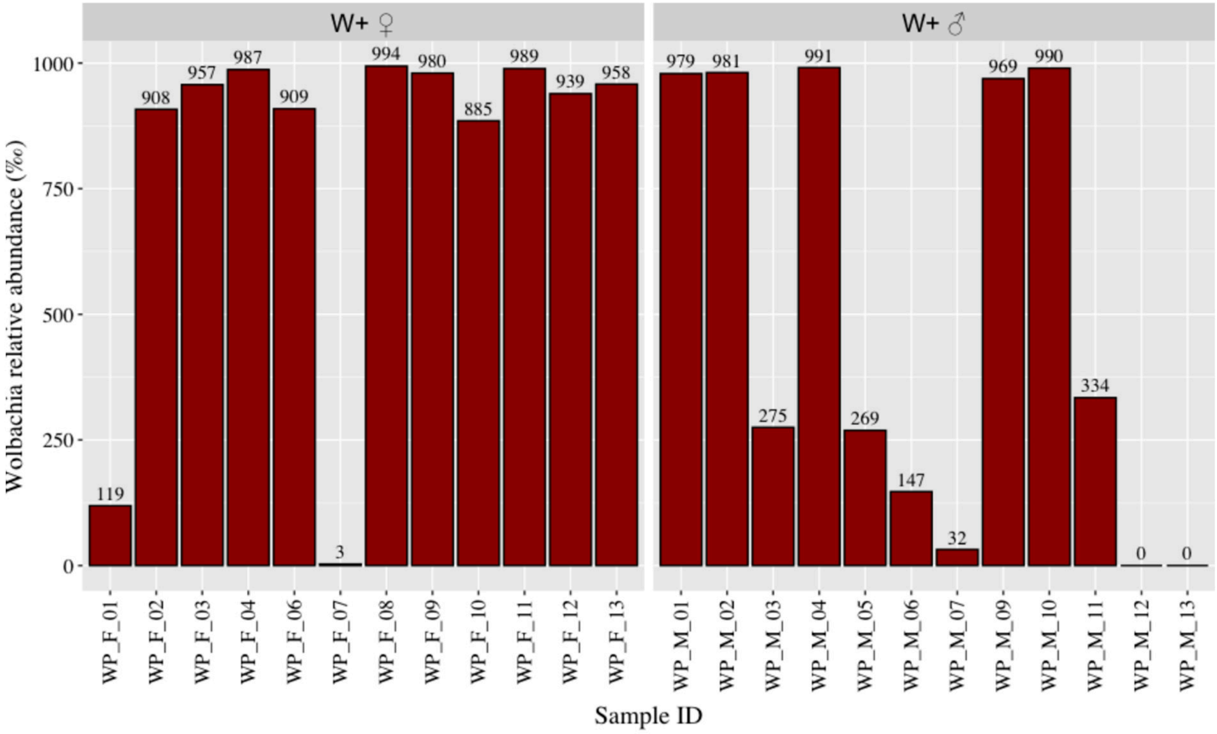

Figure S1. *Wolbachia* distribution among infected female (♀) and male (♂) flies. The *Wolbachia* genus was represented by 8 ASVs. The relative abundance is expressed in per mille.
