# Supplementary material for: Influential Insider: Wolbachia, an Intracellular Symbiont, Manipulates Bacterial Diversity in Its Insect Host
Source: Microorganisms. 2021 Jun 16;9(6):1313. doi: 10.3390/microorganisms9061313 (PMC8234596; doi:10.3390/microorganisms9061313)
Supplement: Supplementary file 1 [file microorganisms-09-01313-s001.zip › Wolbachia_Figure_S3.pdf]

Supporting Information

INFLUENTIAL INSIDER: *WOLBACHIA*, AN INTRACELLULAR SYMBIONT, MANIPULATES BACTERIAL DIVERSITY IN ITS INSECT HOST

Morgane Ourry[1,*], Agathe Crosland[2], Valérie Lopez[2], Stéphane A.P. Derocles[2], Christophe Mougel[1], Anne Marie Cortesero[2] & Denis Poinsot[2]

*** Correspondence:**

Morgane Ourry

morgane.ourry@hotmail.fr

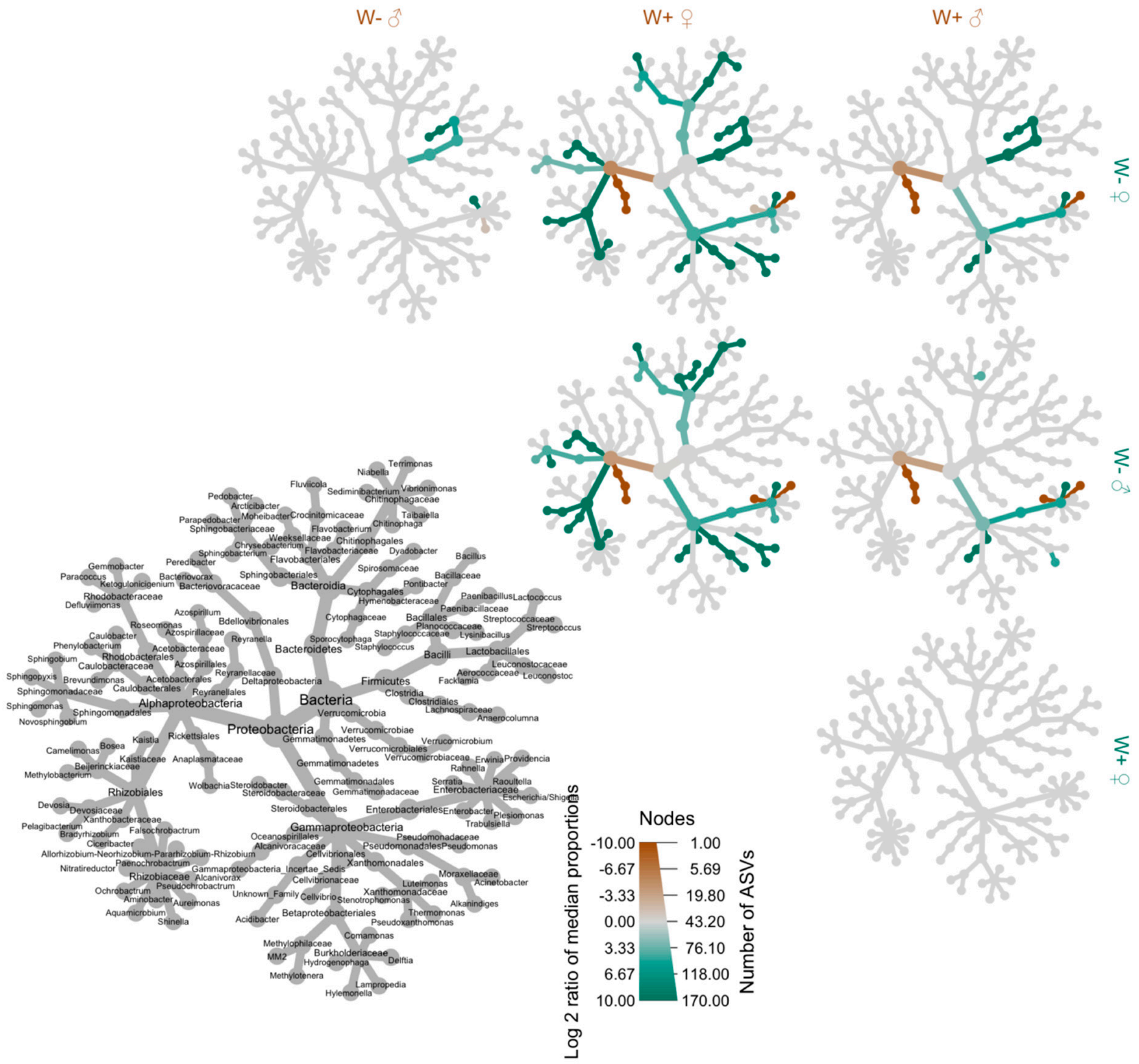

Figure S3. Heat trees comparing taxa relative abundances between female and male *Wolbachia*-free ("W–") and infected ("W+") lines. The color of each taxon represents the log-2 ratio of median relative abundances observed for each treatment. Only significant differences are colored. Taxa colored in green are enriched in the treatment shown in the row while taxa colored in brown are enriched in the treatment shown in the column. The grey tree on the lower left functions as a key for the smaller unlabeled trees.
