# Supplementary material for: Influential Insider: Wolbachia, an Intracellular Symbiont, Manipulates Bacterial Diversity in Its Insect Host
Source: Microorganisms. 2021 Jun 16;9(6):1313. doi: 10.3390/microorganisms9061313 (PMC8234596; doi:10.3390/microorganisms9061313)
Supplement: Supplementary file 1 [file microorganisms-09-01313-s001.zip › Wolbachia_Figure_S4.pdf]

Supporting Information

INFLUENTIAL INSIDER: *WOLBACHIA*, AN INTRACELLULAR SYMBIONT, MANIPULATES BACTERIAL DIVERSITY IN ITS INSECT HOST

Morgane Ourry[1,*], Agathe Crosland[2], Valérie Lopez[2], Stéphane A.P. Derocles[2], Christophe Mougel[1], Anne Marie Cortesero[2] & Denis Poinsot[2]

*** Correspondence:**

Morgane Ourry

morgane.ourry@hotmail.fr

A

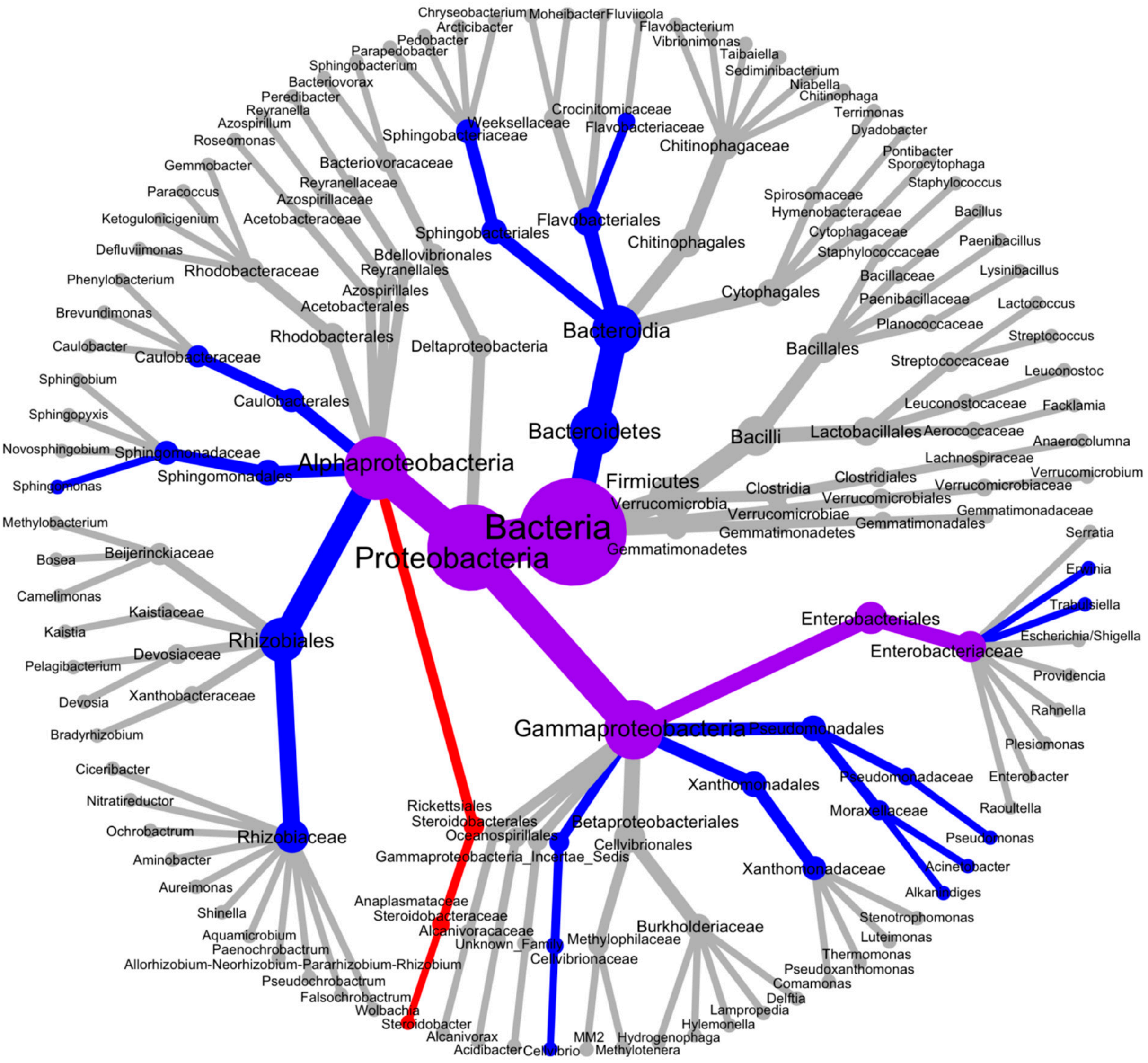

Taxa present in all flies of the W– treatment
Taxa present in all flies of the W+ treatment
Taxa present in all flies of both treatments

B

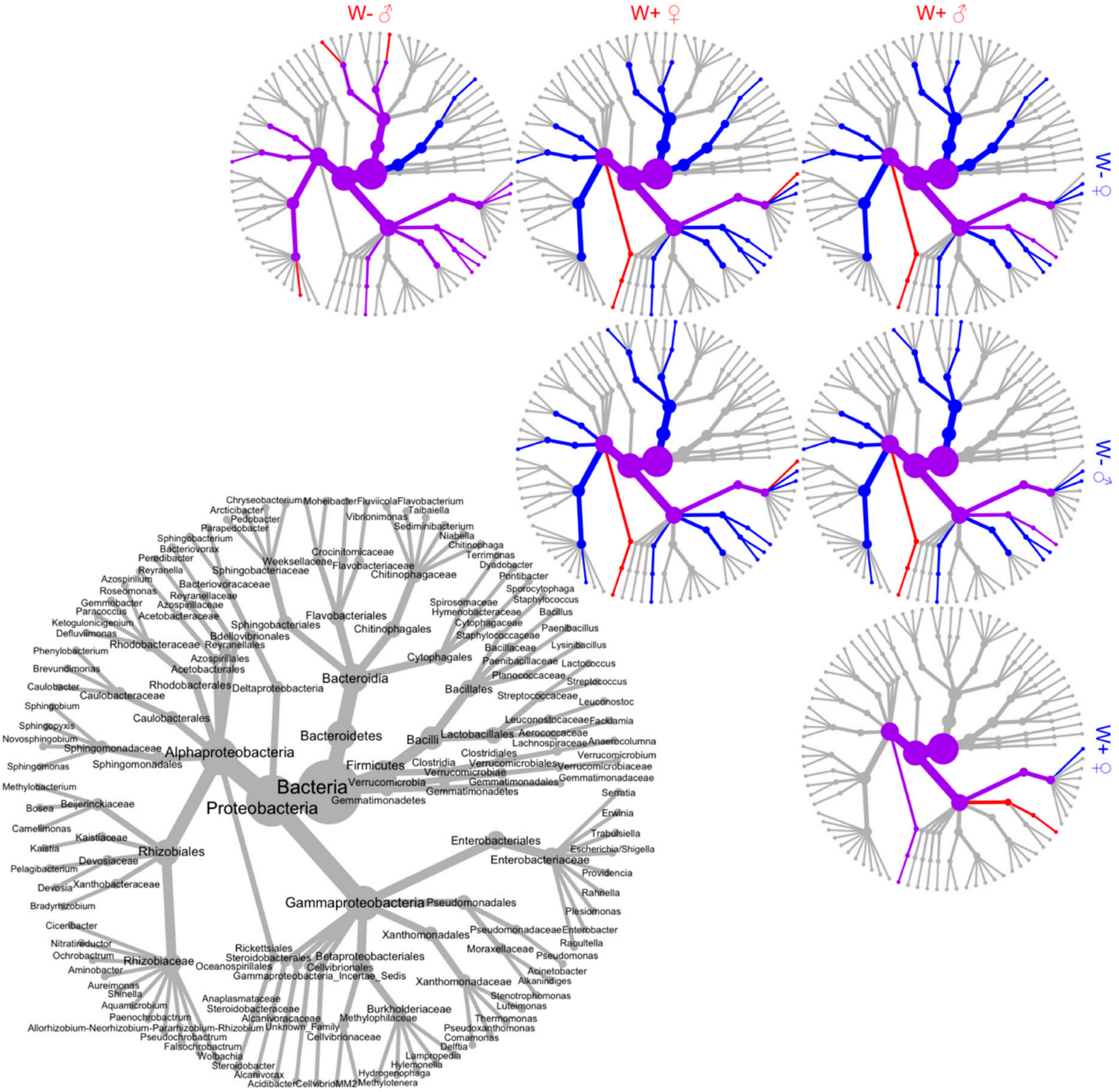

Figure S4. Heat tree comparing taxa presence (A) between *Wolbachia*-free ("W–") and infected ("W+") lines and (B) between female and male W– and W+ lines. (B) Taxa colored in blue are present in all flies of the treatment shown in the row, taxa colored in red are present in all flies of the treatment shown in the column and taxa colored in purple are present in all flies of both treatments shown in the row and the column. The grey tree on the lower left functions as a key for the smaller unlabeled trees.
