# Supplementary material for: Influential Insider: Wolbachia, an Intracellular Symbiont, Manipulates Bacterial Diversity in Its Insect Host
Source: Microorganisms. 2021 Jun 16;9(6):1313. doi: 10.3390/microorganisms9061313 (PMC8234596; doi:10.3390/microorganisms9061313)
Supplement: Supplementary file 1 [file microorganisms-09-01313-s001.zip › Wolbachia_Figure_S6.pdf]

Supporting Information

INFLUENTIAL INSIDER: *WOLBACHIA*, AN INTRACELLULAR SYMBIONT, MANIPULATES BACTERIAL DIVERSITY IN ITS INSECT HOST

Morgane Ourry[1,*], Agathe Crosland[2], Valérie Lopez[2], Stéphane A.P. Derocles[2], Christophe Mougel[1], Anne Marie Cortesero[2] & Denis Poinsot[2]

*** Correspondence:**

Morgane Ourry

morgane.ourry@hotmail.fr

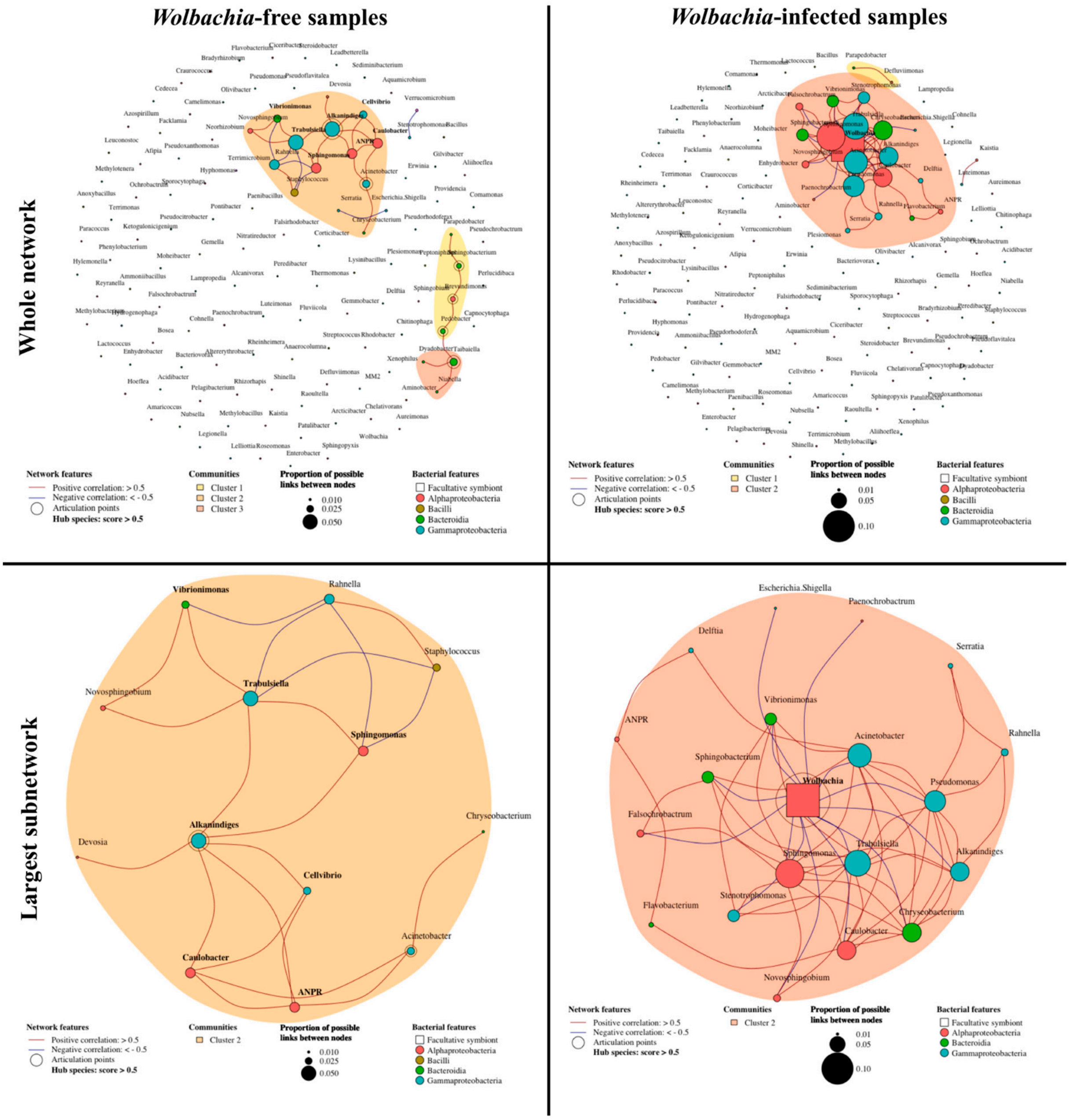

Figure S6. Network and subnetwork of bacterial communities associated to *Wolbachia*-free ("W–") and infected ("W+") lines separately. Taxa represented are genera. Number of vertices: 125 for both W– and W+. Number of edges: 30 for W– and 59 for W+.

ANPR = *Allorhizobium-Neorhizobium-Pararhizobium-Rhizobium*
